# Identification of inflammation-related DNA methylation biomarkers in periodontitis patients based on weighted co-expression analysis

**DOI:** 10.18632/aging.203378

**Published:** 2021-08-04

**Authors:** Pengcheng Wang, Bingbing Wang, Zheng Zhang, Zuomin Wang

**Affiliations:** 1 Department of Stomatology, Beijing Shijitan Hospital, Capital Medical University, Beijing 100038, China; 2 Department of Stomatology, Beijing Chaoyang Hospital, Capital Medical University, Beijing 100020, China; 3 Department of Immunology, School of Basic Medical Sciences, Advanced Innovation Center for Human Brain Protection, Beijing Key Laboratory for Cancer Invasion and Metastasis, Department of Oncology, Capital Medical University, Beijing 100069, China; 4 Department of Periodontology, Tianjin Stomatological Hospital and Tianjin Key Laboratory of Oral Function Reconstruction, Hospital of Stomatology, Nankai University, Tianjin 300041, China

**Keywords:** methylation, CpGs, periodontitis, immune, diagnostic markers

## Abstract

Evidence from past research has shown that DNA methylation plays a key role in the pathogenesis of periodontitis, regulating gene expression levels and thereby affecting the occurrence of various diseases. Three sample sets of methylation data and gene expression data were downloaded from Gene Expression Omnibus (GEO) database. A diagnostic classifier is established based on gene expression data and CpG methylation data. Abnormal expression of immune-related pathways and methyltransferase-related genes in patients with periodontitis was detected. A total of 8,029 differentially expressed CpG (DMP) was annotated to the promoter region of 4,940 genes, of which 295 immune genes were significantly enriched. The CpG sites of 23 differentially co-expressed immune gene promoter regions were identified, and 13 CpG were generally hypermethylated in healthy group samples, while some were methylated in most patients. Five CpGs were screened as robust periodontitis biomarkers. The accuracy in the training data set, the two external verification data sets, and in the transcriptome was 95.5%, 80% and 78.3%, and 82.6%, respectively. This study provided new features for the diagnosis of periodontitis, and contributed to the personalized treatment of periodontitis.

## INTRODUCTION

Periodontitis, which is a chronic multifactorial inflammatory disease related to microbial dysfunction, is characterized by the progressive destruction of periodontal tissues. It is a worldwide public health problem and has a measurable impact on overall health of patients [1]. It is widely believed that the occurrence and development of periodontitis depends on the presence of toxic microorganisms, which are capable of causing periodontitis. Although bacteria are initiator of periodontitis, and response of host to pathogenic infections plays a critical role in the progression of periodontitis [2, 3]. After the onset of periodontitis, periodontitis will progress rapidly with the loss of collagen fibers and attachment to cementum surface, migration of the apex of connective epithelium, deepening of periodontal pockets, and absorption of alveolar bone [4]. If not treated in time, periodontitis will continue to develop, leading to bone destruction, tooth movement and subsequent tooth loss. In the United States, more than half of adults have periodontal disease, and about 10 % have severe periodontal disease and early tooth loss. Traditional periodontal diagnostic parameters include, for example, bleeding detection, clinical attachment degree, plaque index, and x-ray film to assess alveolar bone level [5], which have the advantages of easy-to-use, cost-effectiveness, and less invasiveness. Clinical reading of tooth attachment with periodontal probes and radiological assessments of alveolar bone loss is used to evaluate the extent of damage from past destructive events, and this requires a 2-3 mm threshold change to determine whether a site underwent a significant anatomic event [6]. Therefore, effective molecular diagnostic approach, medical care, and periodontal management are essential to reducing adverse outcomes of periodontitis.

DNA methylation is a heritable epigenetic modification of cells to control gene expression without changing gene sequence [7, 8–11]. With the flexibility of epigenomic modifications, methylation events can respond to nutritional and environmental influences and regulate gene expression patterns accordingly [12, 13], and may also serve as potential biomarkers for early cell transformation [14]. A growing number of articles have focused specifically on epigenetic changes in DNA, highlighting the importance of "epigenetic phenotypes" in many diseases. Methylation is defined as enrichment in the area of CpG island (CGI), representing an area of at least 200 bp, where the ratio of guanine to cytosine is greater than 50%, and CpG ratio is greater than 0.6 [15]. CGIs mainly focuses on gene promoters and is the first candidate for the study of gene expression methylation [16]. Evidence of methylation in affecting gene expression, especially in cancers, has been found. Serum DNA methylation is used as a biomarker for early detection of cancer [17–20]. DNA methylation of specific genes (SEPT9, RASSF1A, APC, GADD45a) has been proposed as a diagnostic and prognostic biomarker for colorectal cancer [21–23] and breast cancer [24].

In this study, abnormal expression of methyltransferase in periodontitis patients was observed, pointing to different methylation patterns in periodontitis patients. Simultaneous activation of multiple immunomodulation-related pathways at the transcriptional level indicated that these abnormal methylation patterns may be related to the dysregulation of immune pathways. The purpose of this study was to integrate high-throughput methylation profile and expression profile data of a large number of patients, so as to study the altered DNA methylation pattern, especially the promoter methylation of inflammation-related genes, between periodontitis patients and healthy people. This study aimed to identify specific DNA methylation sites as potential biomarkers and to establish a diagnostic classifier for periodontitis.

## RESULTS

### The translational disorders in the periodontitis were related to immunity and epigenetic inheritance

**Figure 1:**
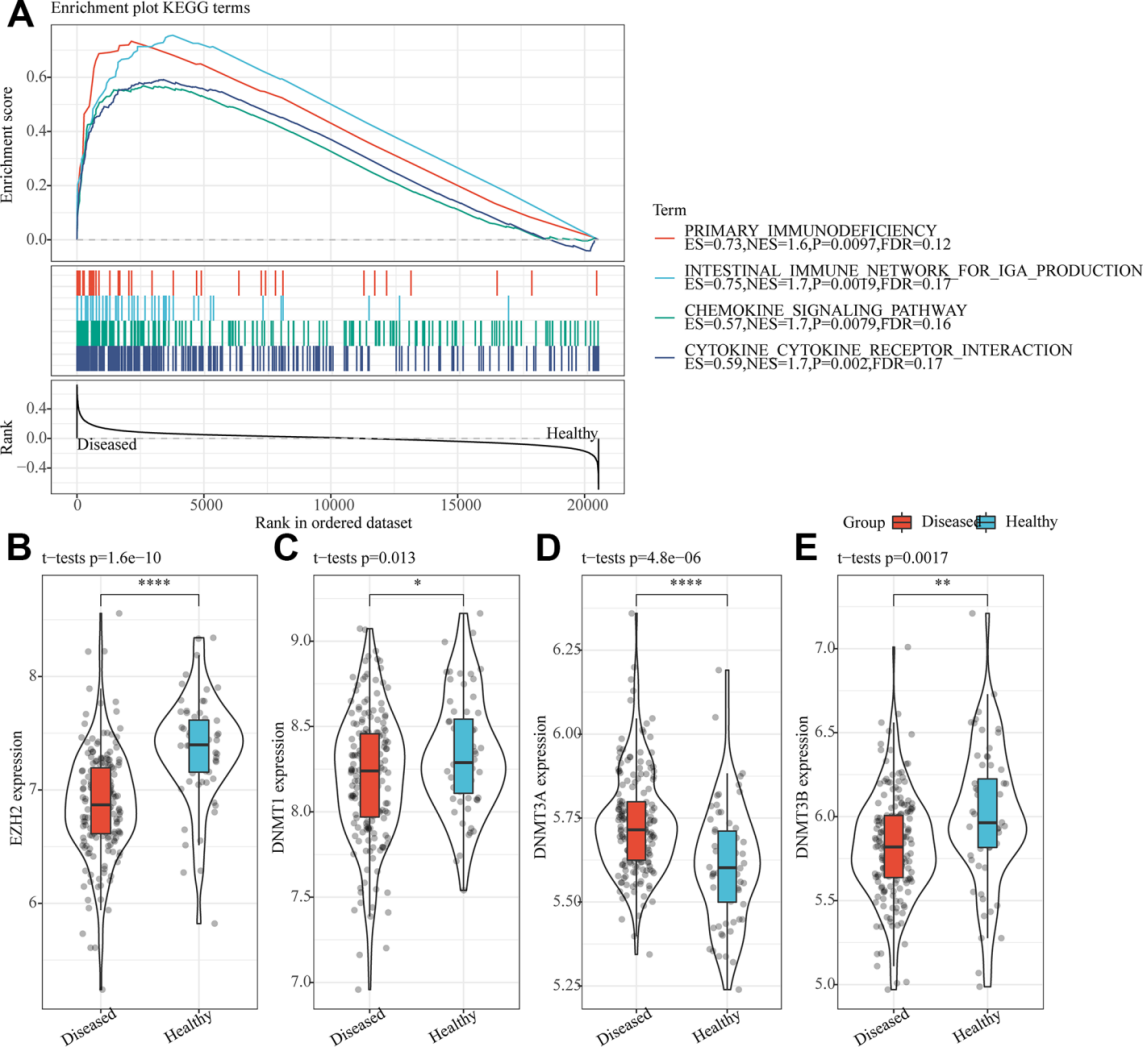
**The translational disorders in the periodontitis were related to immunity and epigenetic inheritance.** (**A**) Differences between four immune-related pathways significantly enriched by GSEA in periodontitis and normal samples. (**B**) Expression of EZH2 in periodontitis and normal samples. (**C**) Expression of DNMT1 in periodontitis and normal samples. (**D**) Expression of DNMT3A in periodontitis and normal samples. (**E**) Expression of DNMT3B in periodontitis and normal samples. ^*^p<0.05, ^**^p<0.01, ^***^p<0.001 and ^****^p<0.0001.

A total of 18 disordered KEGG pathways were identified by GSEA analysis on abnormal KEGG pathway in periodontitis samples (Supplementary Table 1). Noticeably, these 18 pathways were activated in periodontitis, many of them were related to immune regulation-related pathways (Figure 1A), indicating that the abnormal expression of immune-related genes played an important role in the regulation of periodontitis. The expression distribution of methyltransferase-related genes was further analyzed, and it was observed that EZH2, DNMT1 and DNMT3B were significantly upregulated in normal samples (Figure 1B, 1C, 1E), whereas that of DNMT3A was sharply downregulated (Figure 1D), indicating that histone methylation and DNA methylation were abnormal in periodontitis. Gene correlation analysis demonstrated that EZH2 gene was highly positively correlated with DNMT1 and DNMT3N in periodontitis samples (FDR<0.01) (Supplementary Figure 1). These results indicated that apart from the important functions of DNA methylation in the progression of periodontitis, the co-expression relationship between EZH2 and DNMT was related to histone methylation and DNA methylation in periodontitis.

### Identification of DMPs between periodontitis and healthy control samples

**Figure 2:**
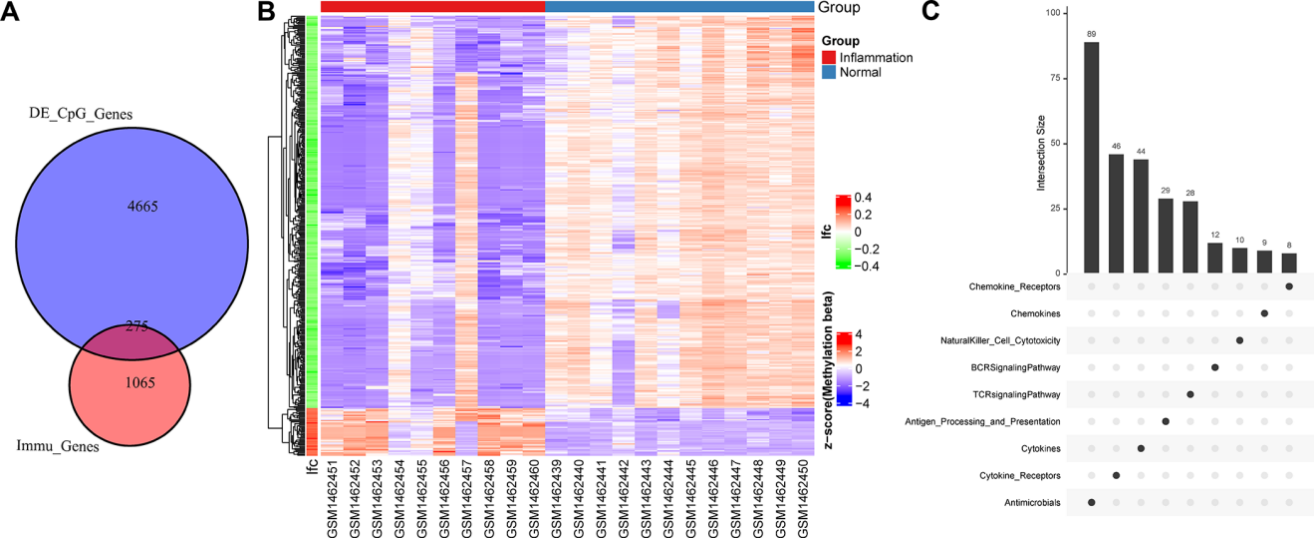
**The relationship between different methylation sites and immune genes.** (**A**) Venn diagram of promoter methylation differential gene and immune gene. (**B**) Heat map of methylation sites of immune gene promoters. The horizontal axis is the sample, the vertical axis is the CpGs, and the color is the methylation level. (**C**) Distribution of immune pathways of differentially promoter methylated immune genes.

Considering that periodontitis transcriptional dysregulation is related to immunity and epigenetics, we analyzed the difference in methylation between normal people and patients with periodontitis, and identified a total of 8029 different CpG sites using Limma (Supplementary Figure 2). By annotating these CpG sites to genes, a total of 4940 genes were obtained, of which 275 were determined to be immune-related genes (Figure 2A). Methylation distribution of 275 immune gene promoter differential CpG sites in periodontitis and normal samples showed that most of these genes were downregulated, which was consistent with the downregulation of methyltransferase at transcription level (Figure 2B). 275 immune genes were mainly distributed in the process of Antimicrobials, Cytokines (Figure 2C), which were consistent with CYTOKINE_CYTOKINE_RECEPTOR_INTERACTION pathways in the transcription activation, suggesting that hypomethylation of promoters of immune-related genes upregulated the expression of immune genes and activated immune-related pathways.

### Weighted co-expression analysis identified immune-related co-expression of DMPs

**Figure 3:**
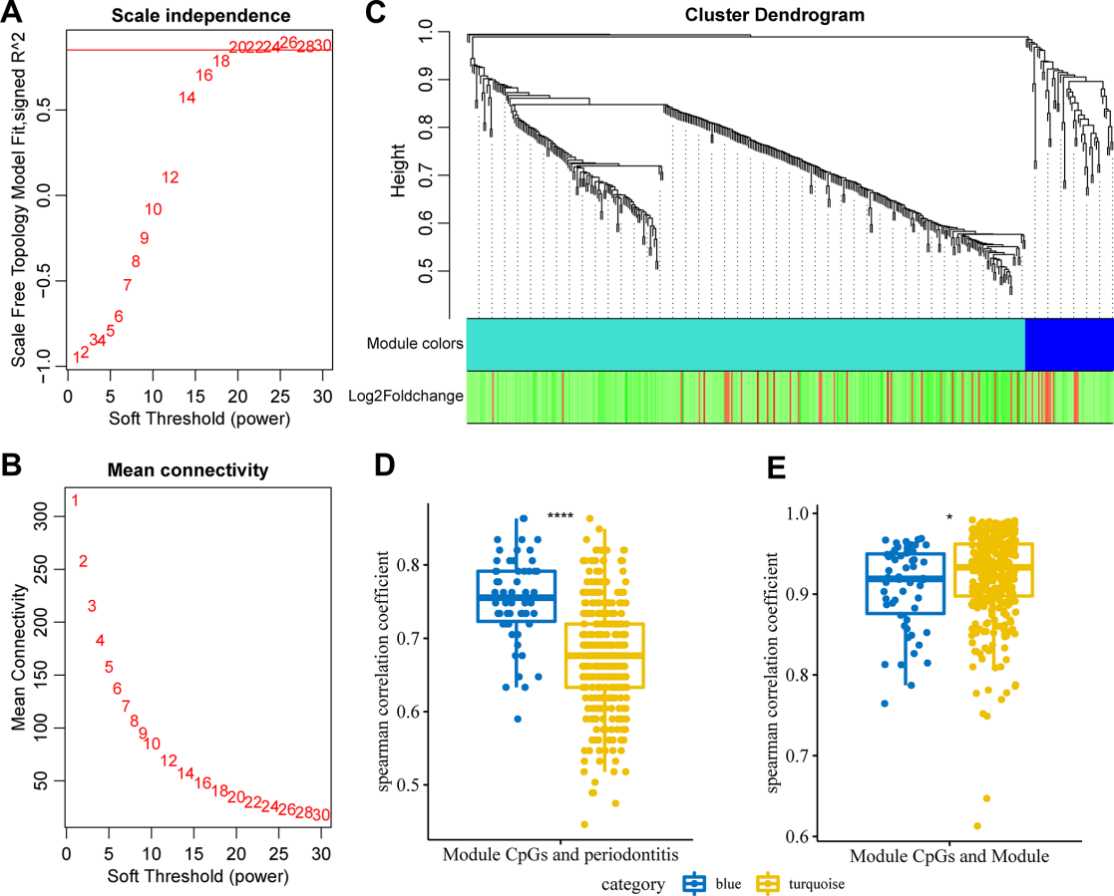
**Weighted co-expression analysis identified immune-related co-expression of DMPs.** (**A**) Analysis of the scale-free fit index for various soft-thresholding powers (β). (**B**) Analysis of the mean connectivity for various soft-thresholding powers. (**C**) Dendrogram of all differentially expressed genes clustered based on a dissimilarity measure (1-TOM). (**D**) The correlation distribution of CpG site methylation in the module and periodontitis. (**E**) The correlation distribution of CpG site methylation in the module and module.

To further screen immune-related co-DMPs, the methylation profiles of 275 immune-related genes at the promoter difference CpG sites were selected, and the co-expression network was constructed using WGCNA. The power of n = 20 (scale-free R^2 = 0.87) was the soft threshold to ensure the scale-free network (Figure 3A, 3B). A total of 2 modules were identified here (Figure 3C). We first calculated the spearman correlation coefficient between CpG site methylation and the occurrence of periodontitis in each module, both modules were found to have a high correlation with periodontitis, and most of the CpGs in the blue module showed a higher correlation with periodontitis (Figure 3D). Furthermore, the correlation coefficient between the eigenvectors of each module and the CpG methylation in the corresponding module was analyzed, and we found that CpGs in blue and turquoise modules were highly correlated with the corresponding feature vectors (Figure 3E). Based on these two methods, we selected CpG loci with higher correlation with the module than the median of the turquoise module and higher correlation with the periodontitis than the median of the blue module, and finally 23 CpG loci were obtained.

### Screening of genomic characteristics of immune-related co-DMPs

**Figure 4:**
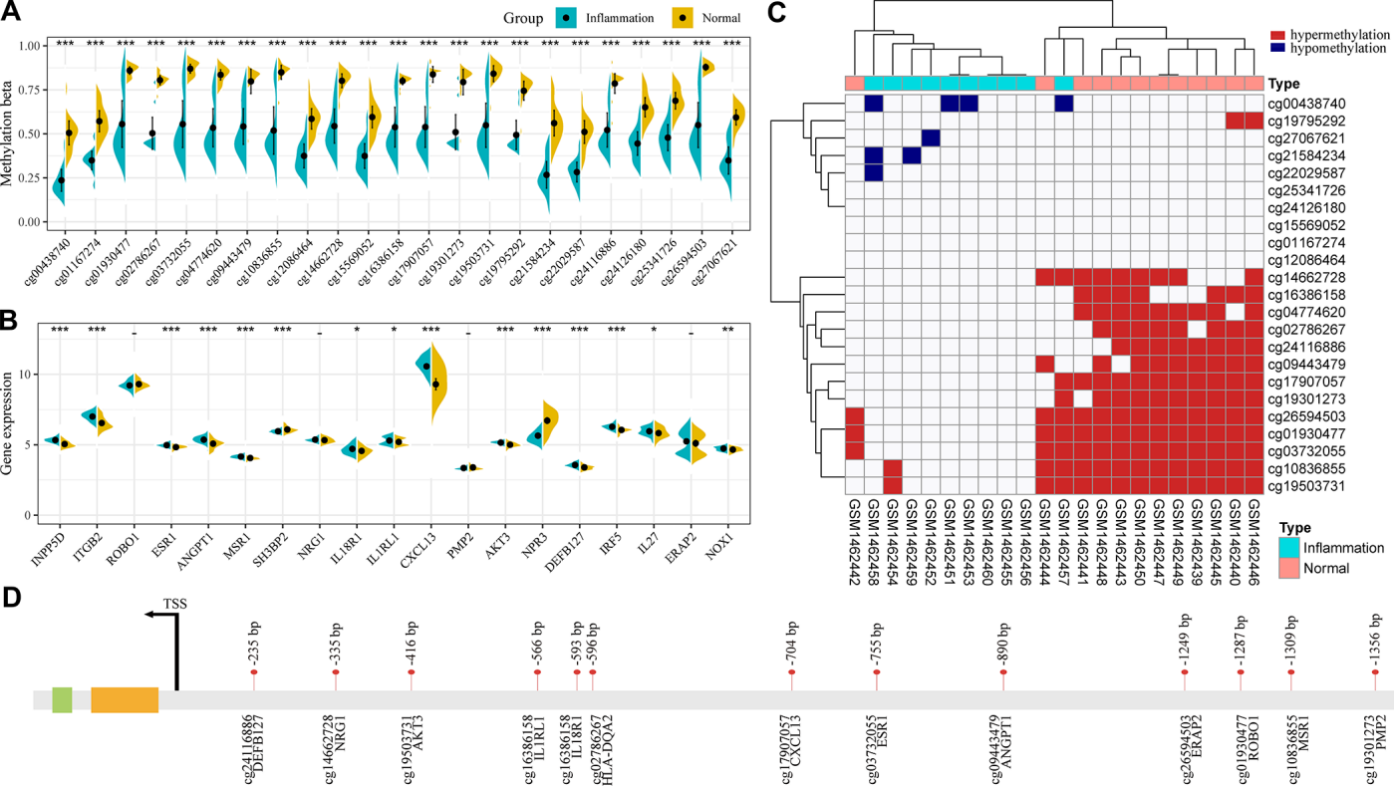
**Genomic characteristics of immune-related co-DMPs.** (**A**) The methylation distribution of 23 co-DMPs in patients and healthy groups. (**B**) The 20 immune genes annotated by 23 co-DMPs were differentially expressed in patients and healthy groups. (**C**) Hypermethylated and unmethylated distributions of 23 co-DMPs in each sample. (**D**) Distribution of 13 potential disease-specific markers in the promoter region.

To further screen immune gene-related CpG markers, the distribution of 23 CpG sites differentially co-expressed in the patients and healthy groups was analyzed (Figure 4A). As expected, these CpG sites were significantly hypomethylated in periodontitis. 23 genes were annotated to the promoter regions of 20 immune genes, and we observed significant differential expression in 15 genes (79%), 13 of them were noticeably overexpressed in the samples of patients with periodontitis (Figure 4B). Furthermore, according to the methylation level of these 23 CpG sites in the samples, those below 0.2 were defined as unmethylated, while those above 0.8 were defined as hypermethylated, and the distribution of hypermethylated and unmethylated CpG sites in each sample was analyzed (Figure 4C). We observed that 13 CPGs (cg01930477, cg02786267, cg03732055, cg04774620, cg09443479, cg10836855, cg14662728, cg16386158, cg17907057, cg19301273, cg19503731, cg24116886, cg26594503) were generally hypermethylated in the healthy group, and some of them were methylated in the majority of patients. The 13 CpG sites was mainly distributed in the upstream of the transcription starting site between 200-1300bp (Figure 4D).

### Construction and testing of diagnostic model

**Figure 5:**
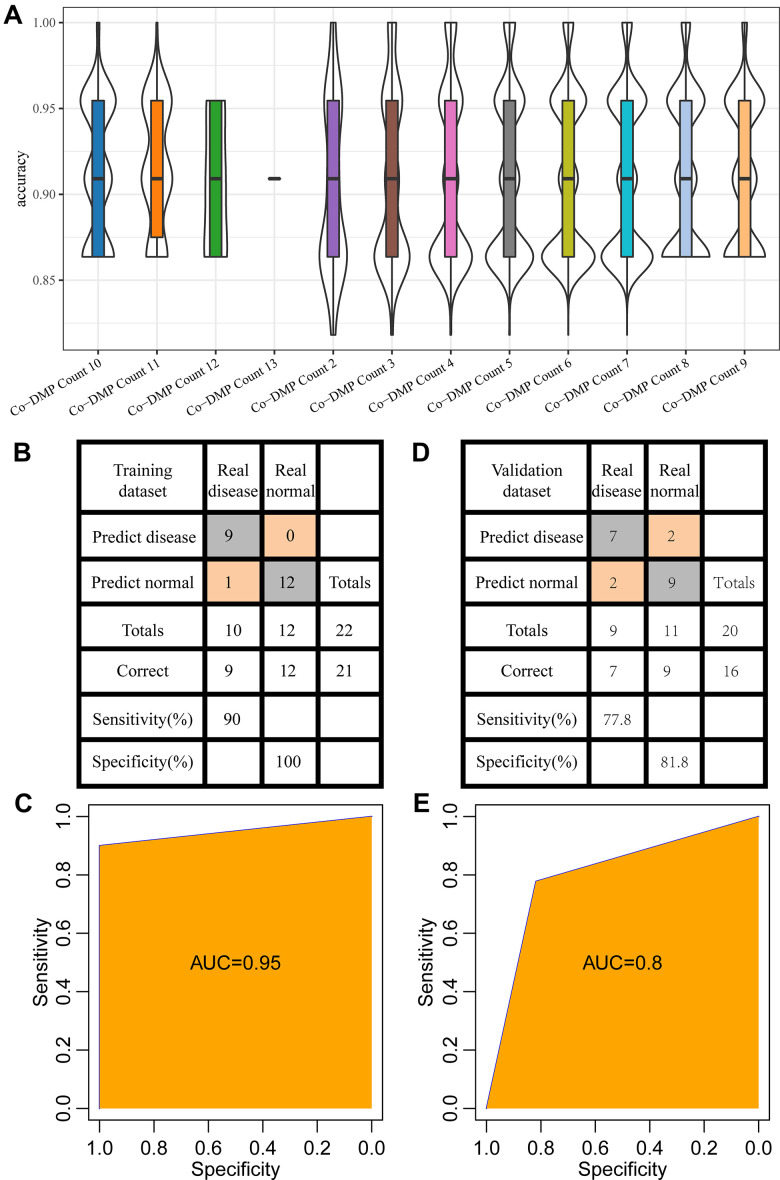
**Establishment of diagnostic model.** (**A**) The accuracy of different combinations of co-DMPs in periodontitis prediction. (**B**) The classification of samples in the training data set by the diagnostic model. (**C**) The ROC curve of the diagnostic model in the training data set. (**D**) The classification of samples in the validation data set by the diagnostic model. (**E**) The ROC curve of the diagnostic model in the validation data set.

In training dataset GSE59932 (N=22, Inflammation=10, Normal=12), to examine the effect of different combinations of diagnostic markers on diagnostic efficiency, all 8178 combinations of 13 Co-DMPs were calculated, and these combinations were used to construct a support vector machine classification model to analyze the prediction accuracy distribution of each combination. The results showed that all have a high prediction accuracy rate, with an average accuracy rate of 90% or higher (Figure 5A). The final 5 CpGs combinations (cg10836855, cg14662728, cg19301273, cg19503731, cg26594503) were determined by stepwise regression and were used to construct the classification model with support vector machine, and the model test was conducted by the ten-fold cross validation method. The results demonstrated that the classification accuracy was 95.5%, as 21 out of 22 samples were correctly classified, and that the sensitivity and specificity of the model were 90% and 100%, respectively (Figure 5B), area under the ROC curve (AUC) was 0.95 (Figure 5C).

Furthermore, the established model was applied to predict the samples in the validation data set (GSE59939, (N=20, Inflammation=9, Normal=11)) to determine the prediction performance of the model. 16 out of the 20 samples were correctly classified, with an accuracy of 80%. The sensitivity and specificity of the model were 77.8% and 81.8%, respectively (Figure 5D), and area under the ROC curve (AUC) was 0.8 (Figure 5E). These results indicated that the established diagnostic prediction model could effectively distinguish the patients with periodontitis from the normal control.

### Validation of the diagnostic model

**Figure 6:**
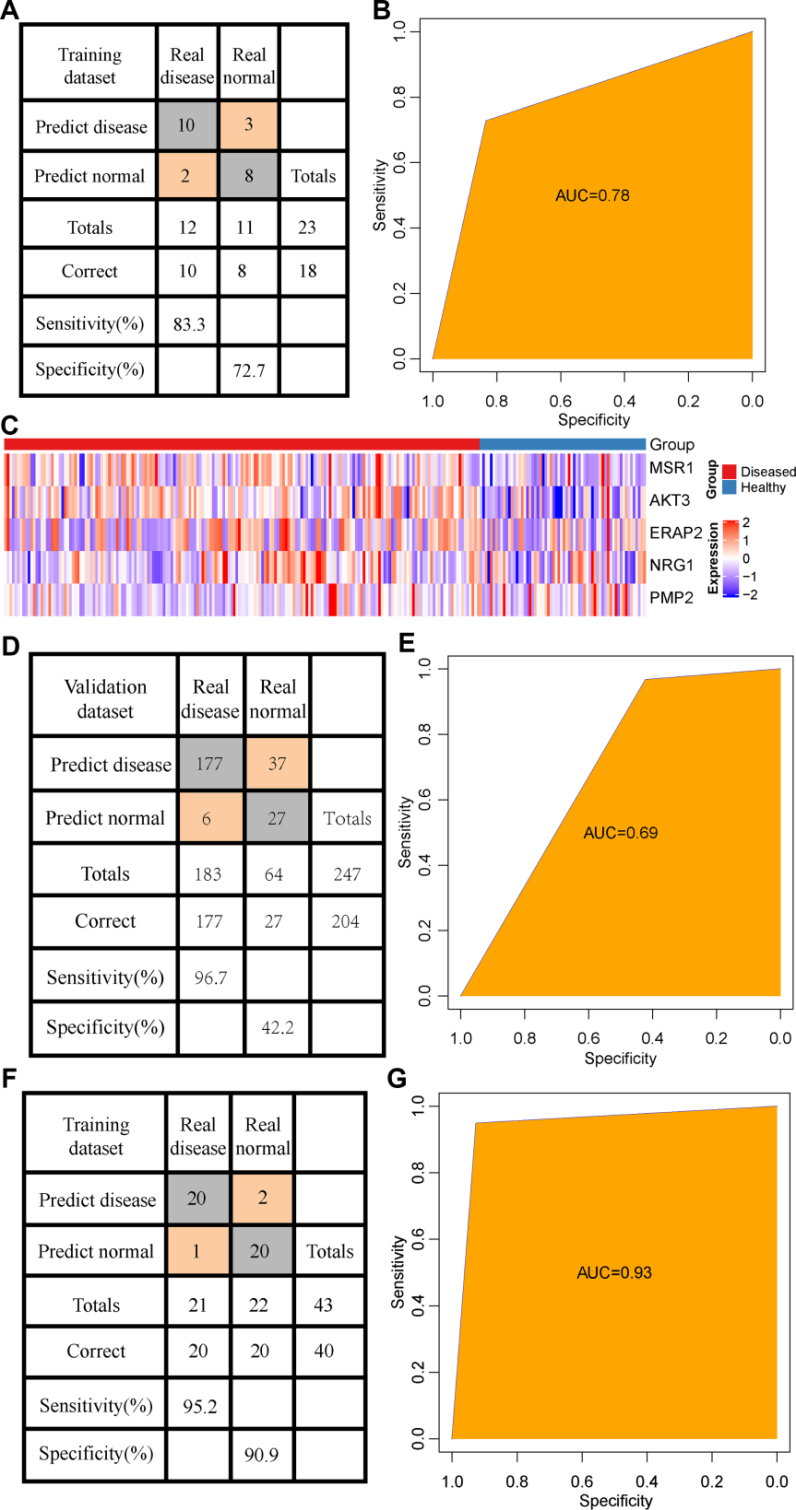
**Validation of the diagnostic model.** (**A**) The classification of samples in the GSE53849 data set by the diagnostic model. (**B**) The ROC curve of the diagnostic model in the GSE53849 data set. (**C**) Heat map of the expression distribution of 5 immune genes annotated by 5 CpG in disease and normal samples. (**D**) The classification of samples in the combined validation data sets (GSE59939 and GSE53849) by the diagnostic model. (**E**) The ROC curve of the diagnostic model in the combined validation data sets (GSE59939 and GSE53849) data set. (**F**) The classification result of the sample by the diagnostic model constructed by immune genes. (**G**) ROC curve of the diagnostic model constructed by immune genes.

A set of independent data set GSE53849 containing 23 samples was selected, and the methylation matrix of 5 CpG was extracted. Here, 8 out of 11 normal samples were predicted as normal, and 10 out of the 12 patients were predicted as periodontitis samples, showing an accuracy rate of 82% (Figure 6A), and area under the ROC curve (AUC) of 0.78 (Figure 6B). In general, the prediction performance of the model was high across the data platform. To analyze the model and immune genetic relationship, 5 CpG loci were annotated to the 5 immune genes (MSR1, NRG1, PMP2, AKT3, ERAP2). The expression profiles of these 5 genes were extracted from the dataset GSE10334. We observed the expression of the 5 genes was significantly higher in patients with periodontitis than healthy controls (Figure 6C). Considering the small number of validation samples, we supplemented and combined two sets of validation data sets (GSE59939 and GSE53849) to form a larger data set incorporating 43 samples in total. Based on this data set, we verified the CpG methylation model again, and found that 20 out of the 22 normal samples were predicted as normal, and that 20 out of 21 patients were predicted as periodontitis patients, showing an accuracy of 93% and the area under the ROC curve of 0.93 (Figure 6D, 6E). Furthermore, the expression profiles of these 5 genes were used to establish a diagnostic model with support vector machine for prediction. Among them, 27 out of 64 normal patients were predicted as normal samples, and 177 out of 183 patients were predicted as periodontitis patients, showing an accuracy rate of 82.6% (Figure 6F) and area under the ROC curve (AUC) of 0.69 (Figure 6G). The above results indicated a high consistency between transcription level and methylation level.

## DISCUSSION

Periodontal disease is caused by bacteria at the tooth biofilm. To eliminate bacteria, immune system cells release substances that may cause inflammation and damage the gums, periodontal ligament, or alveolar bone, thereby causing swelling and bleeding of the gums, which is a sign of gingivitis. Damage from periodontal disease may also lead to tooth relaxation [25]. In this study, differences in gene expression between periodontitis patients and healthy control samples were compared. We found that there were various immune pathway disorders, such as PRIMARY_IMMUNODEFICIENCY, AUTOIMMUNE_THYROID_DISEASE, B_CELL_RECEPTOR_SIGNALING_PATHWAY, etc., in periodontitis patients. This again proved that the inflammation-related pathway was more active in periodontitis [26]. Interestingly, we also found that the TYPE_II_DIABETES_MELLITUS pathway was significantly activated in patients with periodontitis, showing a certain relationship between periodontitis patients and diabetes, and that periodontitis may affect diabetes and diabetes-related complications [27]. Furthermore, by analyzing the expression characteristics of methyltransferase-related genes such as EZH2 and DNMT, we found that these genes were significantly altered in periodontitis patients, and that EZH2 was highly positively correlated with DNMT1 and DNMT3N, indicating that both DNA methylation and histone methylation may play an important role in periodontitis patients [28, 29]. In addition, we preliminarily evaluated methyltransferase molecule EZH2 in periodontitis, and examined its role in PDLSCs, PDLSCs+LPS, PDLSCs+LPS+GSK126. RT-pPCR data showed a lower expression of EZH2 in inflammatory cells, which was consistent with the results of our data analysis and was also confirmed by Western Blot.

DNA methylation is an important epigenetic modification that suppresses gene transcription by inhibiting the binding of specific transcription factors [30]. New evidence showed that epigenetics plays a key role in human pathology, including in inflammation and cancer development. Epigenome is influenced by environmental factors throughout life. Nutritional factors have profound effects on the expression of specific genes through epigenetic modifications and may be passed on to offspring. Many cancers are associated with epigenetic changes, which will lead to changes in the expression of genes involved in cell growth or differentiation. The incidence of autoimmune diseases and tumors increases with age, and epigenetic disorders are considered as a potential explanation for differences in CpG methylation status, single allele silencing, and other epigenetic regulatory mechanisms observed in key inflammatory response genes [31]. In this study, CpGs of 8,029 differentially promoter regions were screened, and 4,940 genes were annotated, among which, immune genes showed stronger promoter methylation differences than random ones, indicating that DNA methylation plays an important role in the transcriptional regulation of immune inflammatory genes in chronic periodontitis.

In addition, if patients with periodontitis are left untreated, periodontitis can continue to develop, leading to bone destruction, tooth movement and subsequent tooth loss. Therefore, early diagnosis of periodontitis and personalized medical intervention are of great significance. Some studies have been conducted to screen biomarkers for periodontitis. JANET S. KINNEY et al. [32] determined that pyridinoline cross-linked carboxy-terminal peptides can predict future disease activity from oral fluids. Giannobile WV screened gingival sulcus fluid for biomarkers indicative of bone loss [33]. Frodge BD et al. [34] identified a bone remodeling biomarker for periodontal disease from saliva. Yoon AJ et al. [35] assessed the association between diabetes and periodontal status and oral inflammatory burden, and identified inflammatory biomarkers in saliva. In this study, 5 CpGs were determined based on the differences in promoter methylation of immune-related genes, and the classifier was constructed and verified. The high accuracy of the classifier in the training set and the external data set indicated that these genes had a strong ability to classify periodontitis and were applicable to different data platforms. Despite reduced accuracy, different methods of data standardization and batch effect as well as environmental factors, different regions, races, diets and some other factors could also the accuracy of external dataset validation. Furthermore, we used the transcriptome data set and the 5 immune genes annotated by the 5 CpGs to build a diagnostic model, which showed an accuracy of periodontitis prediction of 82.6%. These results indicated that these 5 CpGs and 5 immune genes can be used as diagnostic markers for periodontitis, and provide targets and references for clinicians and biological experimentalists.

The 5 CpGs were annotated to 5 immune genes including MSR1, NRG1, PMP2, AKT3, ERAP2. MSR1 is an important marker of macrophages and its abnormal expression is associated with multiple diseases including Porphyromonas gingivalis [36–38]. NRG1, which controls the formation of excitatory and inhibitory synapses in cortical circuits, is a schizophrenia risk gene [39], and is associated with multiple relapsing disorders. In addition, NRG1 plays an important role in parabens of fibroblasts and macrophages [40]. Overexpression of mutant PMP2 also leads to CMT1 phenotype [41], and aberrant AKT3 expression helps form M2 macrophage specificity [42]. These genes are directly or indirectly associated with periodontal disease, and multiple gene expressions will increase the possibility of macrophage abnormalities, suggesting that aberrant methylation of these CpGs may be involved in the development and progression of periodontitis via macrophages.

Although bioinformatics techniques were used to identify potential candidate genes involved in periodontitis in large samples, some limitations of this study should be noted. Firstly, the samples lacked clinical follow-up information, especially some diagnostic details, therefore we did not differentiate diagnostic biomarkers for periodontitis by taking into account factors such as the presence of other patient health conditions. Secondly, the results obtained by bioinformatics analysis alone were not convincing enough, and experimental verification is required to confirm the current results. Therefore, further genetic and experimental studies with larger sample sizes and experimental validation should be performed.

By correlating the expression of inflammation-related genes and the methylation relationship of their promoter regions and combining with the co-expression network, this study identified and screened diagnostic markers for periodontitis. A diagnostic model for the prediction and prevention of periodontitis was established based on the pattern recognition of support vector machine (SVM). We determined the expression and methylation characteristics of epigenetic key genes in periodontitis, and found that these gene promoter methylation was closely related to the occurrence and development of periodontitis. Although our gene expression profile still lacks high specificity required for immediate diagnostic application, CpG methylation in oral samples could predict periodontitis with high accuracy (AUC = 0.95), providing a target and reference for clinicians and biological experimentalists.

## MATERIALS AND METHODS

### Data collection

**Figure 7:**
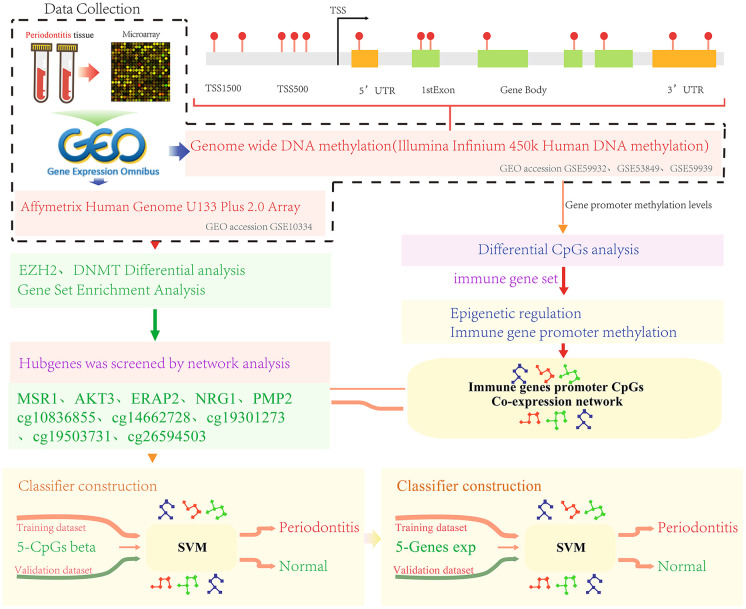
Work flow chart.

We screened three sets of gene methylation data and one set of gene expression data from the Gene Expression Omnibus (GEO) database (http://www.ncbi.nlm.nih.gov/geo/) [43]. The methylation data came from the GPL13534 platform (Illumina HumanMethylation450 BeadChip). The gene expression profile data was from GPL570 platform (Affymetrix Human Genome U133 Plus 2.0 Array) numbered GSE10334 [44]. The data set contained a total of 183 periodontitis patient samples and 64 healthy control samples. The sample distribution of each data set is shown in Table 1. The work flow chart is shown in Figure 7.

**Table 1:** Sample distribution of data set.

**GEO Accession**	**No. of Normal**	**No. of Periodontitis**
GSE59932	12	10
GSE59939	11	9
GSE53849	11	12
GSE10334	64	183

### Methylation data processing

The methylation β values of the standardized CpG sites were downloaded, and the missing values of CpG sites greater than 20% in each sample were removed. The missing value completion was performed using the R package impute [45]. Probes binding to sex chromosomes, cross-hybridizing to multiple locations, or targeting a single-nucleotide polymorphism (SNP) were removed, according to previous annotation [46, 47]. By referring to the processing method of Zhang et al. [48], the methylation site of the non-promoter region was further removed, resulting in 232189 probes for DNA methylation analysis. All analysis was performed using β values to improve the statistical calculation of differential methylation [49, 50]. β values are also included in the tables for biological interpretation.

### Gene chip data processing

For gene expression data, we first downloaded the standardized chip data. R package hgu133plus2.db was used for probe annotation. Probes that match multiple genes were removed. When multiple probes matched to one gene, the median of these probes were used as the expression value of the modified gene. Finally, the expression profiles of 20549 genes were obtained. In addition, we also downloaded 2498 immune genes from InnateDB [51].

### Identification of differential methylation

The R software package limma [52] was used to detect different methylation sites between periodontitis patients and normal samples. Difference multiple greater than 20% and FDR <0.05 was the threshold to include more CpG sites with statistical differences.

### Gene set enrichment analysis

Gene Set Enrichment Analysis(GSEA) [53] was performed by the JAVA program (http://software.broadinstitute.org/gsea/downloads.jsp) using the MSigDB [54] C2 Canonical pathways gene set collection with 1320 gene sets. After performing 1000 permutations, significantly enriched gene set was defined as having a p value less than 0.05.

### Co-expression network construction

CpG methylation data profile of DMPs was assessed to evaluate whether the samples and CpGs included were qualified. Then, we used the weighted gene co-expression network analysis (WGCNA) [55] package in R to construct scale-free co-expression network for the DMPs. Pearson's correlation matrices and average linkage method were performed for all pair-wise CpGs. Then, a weighted adjacency matrix was constructed using a power function A*_mn_* = |C*_mn_*|*^β^* (C*_mn_* = Pearson's correlation between CpG m and CpG n; A*_mn_* = adjacency between CpG m and CpG n). β was a soft-thresholding parameter that could emphasize strong correlations between CpGs and penalize weak correlations. After choosing the power of β, the adjacency was transformed into a topological overlap matrix (TOM) to measure the network connectivity of a CpG, which is defined as the sum of its adjacency to all other CpGs for network CpG ration. The corresponding dissimilarity (1-TOM) was also calculated. To classify CpGs with similar expression profiles into CpG modules, average linkage hierarchical clustering was conducted according to the TOM-based dissimilarity measured with a minimum size (CpG group) of 30 for the CpGs dendrogram. To further analyze the module, we calculated the dissimilarity of module eigenCpGs, determined a cut line for module dendrogram and merged some modules.

### Construction of diagnostic prediction model and evaluation of model prediction performance

A diagnostic prediction model was constructed using feature genes based on support vector machine (SVM) classification [56]. SVM is a supervised learning model of machine learning algorithm, and can analyze data and identify patterns. A support vector mechanism creates a hyperplane in a high or infinite dimensional space and can be used for classification and regression. All the samples were randomly and uniformly divided into training data set and verification data set. The model was constructed in the training data set, and the classification ability of the model was verified by ten-fold cross validation method. The established model was then used to predict the samples in the validation data set. The predictive performance of the model was assessed with the area under the ROC curve (AUC), and the sensitivity and specificity of the model for predicting periodontitis were analyzed.

### Clinical effectiveness of the model

Two sets of Illumina HumanMethylation450 BeadChip platform data sets GSE59939 [57] and GSE53849 were selected as independent external verification data sets. After downloading the standardized data, the methylation level of characteristic CpGs was extracted and substituted into the model to assess the prediction ability of the model. Furthermore, a set of expression profile data set GSE10334 [58] was used to extract the expression profile of immune genes from the characteristic CpGs annotation to the promoter. A diagnostic model was established to distinguish normal healthy samples from periodontitis.

## Supplementary Material

Supplementary Figures

Supplementary Table 1
