# Supplementary material for: Identification of inflammation-related DNA methylation biomarkers in periodontitis patients based on weighted co-expression analysis
Source: Aging (Albany NY). 2021 Aug 4;13(15):19678–95. doi: 10.18632/aging.203378 (PMC8386560; doi:10.18632/aging.203378)
Supplement: Supplementary Figures [file aging-13-203378-s001.pdf]

SUPPLEMENTARY FIGURES

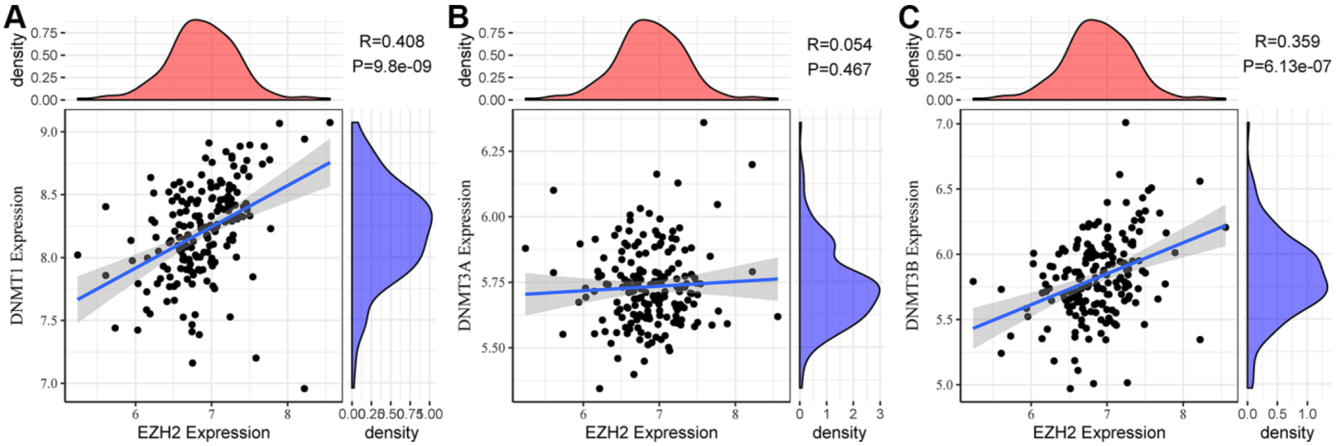

Supplementary Figure 1. Correlation between EZH2 and DNMT expression. (**A**) Correlation between EZH2 and DNMT1 expression. (**B**) Correlation between EZH2 and DNMT3A expression. (**C**) Correlation between EZH2 and DNMT3B expression.

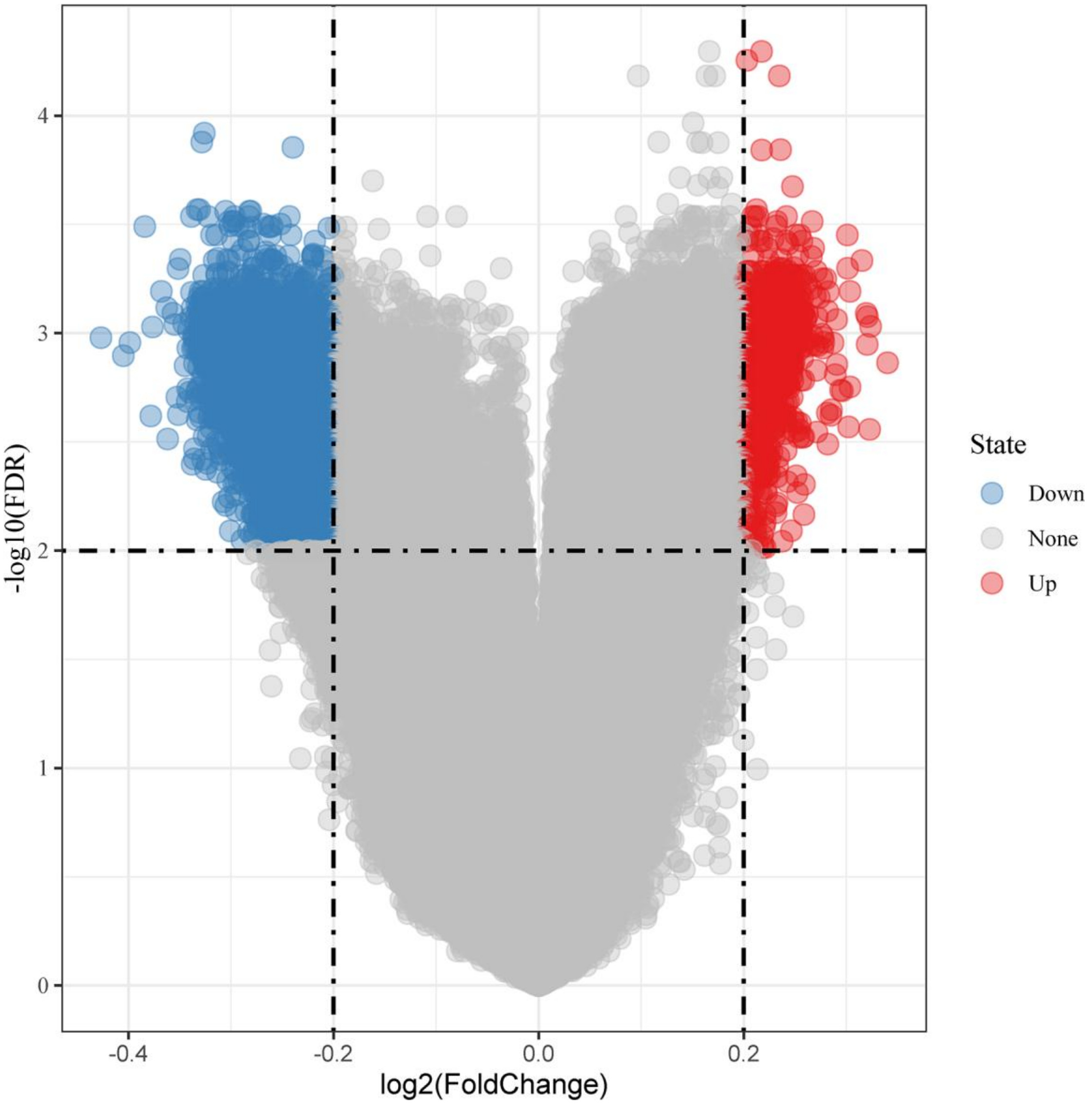

Supplementary Figure 2. Volcanic map of methylation difference, x axis is the difference multiple, y axis is the difference significance, red is the up-regulation of DMPs, green is the down-regulation of DMPs.
