# Supplementary material for: Identification of inflammation-related DNA methylation biomarkers in periodontitis patients based on weighted co-expression analysis
Source: Aging (Albany NY). 2021 Aug 4;13(15):19678–95. doi: 10.18632/aging.203378 (PMC8386560; doi:10.18632/aging.203378)
Supplement: Supplementary Table 1 [file aging-13-203378-s002.pdf]

SUPPLEMENTARY TABLE

Supplementary Table 1. 18 disordered KEGG Pathways.

Term	ES	NES	NP	FDR	FWER
KEGG_PRIMARY_IMMUNODEFICIENCY	0.7327	1.6282	0.0097	0.1174	0.549
KEGG_PRION_DISEASES	0.635	1.632	0.0099	0.1213	0.538
KEGG_LEISHMANIA_INFECTION	0.711	1.6323	0.01	0.1303	0.538
KEGG_TOLL_LIKE_RECEPTOR_SIGNALING_PATHWAY	0.5487	1.6037	0.0361	0.1374	0.608
KEGG_CELL_ADHESION_MOLECULES_CAMS	0.5644	1.6349	0.0117	0.1376	0.531
KEGG_AUTOIMMUNE_THYROID_DISEASE	0.6821	1.6422	0.0118	0.1397	0.507
KEGG_HYPERTROPHIC_CARDIOMYOPATHY_HCM	0.4577	1.5942	0.0279	0.1403	0.625
KEGG_HEMATOPOIETIC_CELL_LINEAGE	0.6315	1.6439	0.004	0.1503	0.501
KEGG_B_CELL_RECEPTOR_SIGNALING_PATHWAY	0.5668	1.5776	0.0214	0.152	0.661
KEGG_TYPE_II_DIABETES_MELLITUS	0.4272	1.5695	0.0319	0.1547	0.682
KEGG_CHEMOKINE_SIGNALING_PATHWAY	0.5679	1.6508	0.0079	0.1569	0.487
KEGG_GRAFT_VERSUS_HOST_DISEASE	0.7032	1.5577	0.0385	0.1638	0.7
KEGG_COMPLEMENT_AND_COAGULATION_CASCADES	0.6361	1.7256	0.0039	0.1669	0.303
KEGG_GLYCOSAMINOGLYCAN_BIOSYNTHESIS_KERATAN_SULFATE	0.6808	1.5406	0.0261	0.1709	0.731
KEGG_INTESTINAL_IMMUNE_NETWORK_FOR_IGA_PRODUCTION	0.7548	1.6661	0.0019	0.1728	0.446
KEGG_CYTOKINE_CYTOKINE_RECEPTOR_INTERACTION	0.5915	1.6524	0.002	0.1738	0.484
KEGG_LEUKOCYTE_TRANSENDOTHELIAL_MIGRATION	0.5746	1.7432	0.002	0.1797	0.271
KEGG_VASCULAR_SMOOTH_MUSCLE_CONTRACTION	0.373	1.5017	0.0339	0.1858	0.797
